# An efficient General Transit Feed Specification (GTFS) enabled algorithm for dynamic transit accessibility analysis

**DOI:** 10.1371/journal.pone.0185333

**Published:** 2017-10-05

**Authors:** S. Kiavash Fayyaz S., Xiaoyue Cathy Liu, Guohui Zhang

**Affiliations:** 1 Department of Civil & Environmental Engineering, University of Utah, Salt Lake City, UT, United States of America; 2 Department of Civil & Environmental Engineering, University of Hawaii at Manoa, Holmes, Honolulu, HI, United States of America; Beihang University, CHINA

## Abstract

The social functions of urbanized areas are highly dependent on and supported by the convenient access to public transportation systems, particularly for the less privileged populations who have restrained auto ownership. To accurately evaluate the public transit accessibility, it is critical to capture the spatiotemporal variation of transit services. This can be achieved by measuring the shortest paths or minimum travel time between origin-destination (OD) pairs at each time-of-day (e.g. every minute). In recent years, General Transit Feed Specification (GTFS) data has been gaining popularity for between-station travel time estimation due to its interoperability in spatiotemporal analytics. Many software packages, such as ArcGIS, have developed toolbox to enable the travel time estimation with GTFS. They perform reasonably well in calculating travel time between OD pairs for a specific time-of-day (e.g. 8:00 AM), yet can become computational inefficient and unpractical with the increase of data dimensions (e.g. all times-of-day and large network). In this paper, we introduce a new algorithm that is computationally elegant and mathematically efficient to address this issue. An open-source toolbox written in C++ is developed to implement the algorithm. We implemented the algorithm on City of St. George’s transit network to showcase the accessibility analysis enabled by the toolbox. The experimental evidence shows significant reduction on computational time. The proposed algorithm and toolbox presented is easily transferable to other transit networks to allow transit agencies and researchers perform high resolution transit performance analysis.

## Introduction

Public transit accessibility is becoming an increasingly important topic among researchers and transit agencies for two main reasons. First, enhanced transit accessibility encourages multimodal and active transportation (i.e. walking and biking), and reduce personal vehicular trips. Consequently, it will lead to improved public health and reduction in green-house gas emissions [1–4]. Second, transit-dependent populations rely heavily on public transit for accessing essential services (e.g. job, school, health care, and grocery). Transit accessibility thus plays a critical role in achieving social equity [5–7]. Consequently, transit accessibility analysis can guide decision makings related to transportation investment and land use development [8].

Transit accessibility is defined as the ease of travel for an individual to reach a desired destination via public transit. Previous studies have identified several transit accessibility measures which can be categorized on the basis of whether travel time is taken into account. The *travel time discretionary* measures, which do not consider travel time, focus on service coverage, frequency, and comfort of service. However, overlooking the impact of travel time, which is a major contributing factor for evaluating the ease and feasibility of transit use, tends to overestimate the accessibility [9]. Consequently, the *travel time dependent* measures, accounting for the travel time between origin and destination on top of other miscellaneous factors (e.g. service coverage), have been gaining popularity in recent years [10]. Most of the relevant studies using the travel time dependent measures focused on transit accessibility for specific time-of-day (e.g. peak hour), yet ignored the temporal fluctuation in travel time throughout the day due to transit schedule variation [11]. The drawback for such analysis is that it might lead to an over-optimistic evaluation in transit accessibility due to the frequent service during peak hours. It is thus necessary to track such measures in both spatiotemporal dimensions.

Computing transit accessibility measure in spatiotemporal dimensions requires the calculation of travel time across all transit stations pairs at any given time-of-day. This process is computationally expensive and time consuming using the readily available commercialized software which makes it almost impossible for normal computers to perform [12]. Three main factors are causing this issue. First, the commercialized software packages often use high-level programming languages that increase the computation time. Second, the current algorithms used are developed to find the shortest path between any specific origin-destination (OD) pair [13]. Such algorithms will iterate until the shortest path is found between all OD pairs. Their efficiency thus degrades as network size grows. Third, the feasibility of transit trips from users’ perspective is overlooked. The existing algorithms only minimize (instead of minimizing and limiting) the number of transfers, walking distance, and travel time when searching for the shortest path. Yet this may result in unrealistic routing. For example a route with 5 transfers and 2 mile walking distance is certainly not feasible for transit users. In addition, past research shows that over 90% of transit trips involve only one or two transfers [14].

The contributions of this paper are thus threefold. First, we introduce an innovative algorithm that can significantly decrease the computation required for calculating transit accessibility in both spatial and temporal dimensions. Second, we implement the algorithm in C++ and develop an open-source toolbox that will take advantage of open data (i.e. GTFS and census data) to track the patterns of various transit accessibility measures. Third, we identify the service gap through effective interpretation of the accessibility results.

The rest of the paper is structured as follows. We will review existing studies on transit accessibility and optimal path finding in transit network. This is followed by a brief description of GTFS data. *Methodology* section presents the proposed all-pairs shortest path algorithm design that is implemented in our toolbox to measure transit accessibility, as well as a discussion on travel time matrix construction and its influence on accessibility fluctuation. Then algorithm evaluation and accessibility interpretation are presented through an application demo using the St. George’s transit network. Implications and conclusions are discussed at the end.

## Literature review

### Transit accessibility

Transit accessibility consists of two core elements: activity element and transportation element [15–16]. The activity element reflects the potential opportunities available at a destination and is usually measured by population density, job density, and/or service/facility availability at the destination. The transportation element reflects the ease of travel and is affected by the spatial and temporal coverage of transit, cost of travel (e.g. travel time), and comfort of service.

Several *travel time dependent* accessibility measures have been developed to date such as competition measures [17–19], constraints-based measures [18, 20–21], composite measures [22], cumulative and gravity-based measures [10–11, 23]. The latter two are the most widely used ones [24–29]. Cumulative measures are based on the number of potential opportunities to be reached within certain cost (e.g. travel time) threshold [20, 30–32], and can be expressed as:

$$A_{ic}=\sum_{j=1}^{J}B_{ij}*a_{j}$$ (1)

where *A*_*ic*_ is the cumulative accessibility measure at location *i*, *a*_*j*_ represents the potential opportunities at location *j*, and *B*_*ij*_ is a binary value, with 1 indicating that location *j* can be reached within a predetermined threshold (e.g. within 1-hour travel time window) and 0 otherwise. This measure assumes that a destination is reachable if and only if the impedance of reaching it is lower than the threshold. As a result, two destinations with the same potential opportunities would have the same impact on the measure as long as the impedance of reaching them are both within the threshold. Additionally, if the travel time to a desired destination is slightly outside the predetermined threshold, then this destination is deemed as inaccessible.

Gravity-based measures attempt to address this single-threshold deficiency by weighting the potential opportunities that can be reached based on a cost function (e.g. travel time) [20, 30, 33–34]. The general form is:

$$A_{ig}=\sum_{j=1}^{J}O_{j}*f(C_{ij})$$ (2)

where *A*_*ig*_ is the gravity-based accessibility measure at location *i*, *O*_*j*_ is the potential opportunities at location *j*, and *f (C*_*ij*_*)* is the impedance or cost function (e.g. travel time) for travelling between *i* and *j* via public transit. The main challenge of this method is the need of developing an impedance function between all OD pairs, other than estimating the number of potential opportunities at each location [35]. Gravity-based measure is able to account for spatial coverage, service frequency, destination attractiveness, and travel time between origins and destinations. By adding the temporal dimension to the gravity-based measure, it provides the most comprehensive picture of transit accessibility.

Weighted Average Travel Time (WATT) is in nature a gravity-based accessibility measure that weights travel times based on the attractiveness (potential opportunities) of destinations. According to Cao et al. [36], the WATT between stations can be described as:

$${WATT}_{i}=\frac{\sum_{j=1}^{J}M_{j}*{tt}_{ij}}{\sum_{j=1}^{J}M_{j}}$$ (3)

where *WATT*_*i*_ is the weighted average travel time of station *i*, also referred to as location indicator [37–38]. *M*_*j*_ is the potential opportunities (e.g. population density) of station *j*, *tt*_*ij*_ is the travel time (including egress, ingress, waiting, and transfer time) via public transit from station *i* to station *j*, and *J* is the total number of stations within transit network. WATT is based on gravity-like interaction pattern between locations [39]—increase in potential opportunity (gravity) and decrease in travel time (distance) will increase the accessibility (gravity force) between two stations (masses). WATT is intuitive to interpret. For example, *WATT*_*1*_ = 60 minutes, indicates the average travel time from station 1 to all the other stations is 60 minutes. Calculating WATT for all time-of-day will provide a comprehensive transit accessibility measure that captures the temporal variation in services.

The major drawback of past studies, as Farber et al. [12] mentioned, is the missing piece of tracking the temporal fluctuation in travel time throughout the day due to computational intensity. This directly results in an over/under-estimation in transit accessibility [12, 14, 27, 40]. Farber et al. [12] reported that the calculation of travel time between all stations for all time-of-day for Salt Lake City network with 1,400 stations, and 100 transit routes would take approximately 60 days on a quad-core machine in ArcGIS.

### Transit optimal path finding

Shortest path algorithms have been widely used in transportation to find the minimum travel time between OD pairs. The classic Dijkstra algorithm [41] finds the feasible path between given origin node to all destinations in an oriented graph. Later on, various speed-up techniques and algorithms have been developed to reduce the time complexity of the shortest path algorithm including heuristic algorithms [42–44], parallel computing [45], fuzzy algorithms [46–47], re-optimizing algorithms for dynamic networks [48], and timetable information system [49–51].

Within the context of public transit network, minimizing travel time is not the only objective of an optimal path. Instead, it is a multi-objective optimization problem that aims at minimizing travel time, number of transfers, walking distance, etc. Tan et al. [52] introduced reasonable paths finding algorithm (multi-objective) that constraints on travel time and walking distance. K shortest paths algorithms strive to address the multi-objective path finding problem by giving users the freedom to choose their desired path among several shortest (feasible) paths alternatives between a given OD pair [53–56]. However, both methods are only suitable when finding the shortest paths between a given OD pair. The optimal path finding algorithms (e.g. Dijkstra) are more efficient comparing with K shortest algorithms as they only generate one shortest path result. Time complexity of Thorup shortest path algorithm [57], as one of the fastest single-pair shortest path algorithms introduced for directed graphs with nonnegative weights [58] to date, is *O (E + V log (log (V)))*, where E and V represent the number of edges (routes between each two consecutive stations) and vertices (stations), respectively. Yet the time complexity of single-pair shortest path algorithms increase significantly when implemented onto all OD pairs. All-pairs shortest path algorithm introduced by Pettie [44] has the time complexity of *O (EV + V*^*2*^ *log (log (V)))*. The computation time grows as the network (number of stations and routes) expands. In addition, the edge weights are fixed and known in these algorithms. However, in transit network, the edge weight represents travel time between vertices, which changes throughout the day as the transit schedule and waiting time varies. Thus, an additional step of building the transit graph for each time-of-day is necessary for the all-pairs shortest path algorithm and for transit accessibility analysis.

### GTFS

GTFS was created in 2005 by Google and TriMet for transit agencies to describe their schedules, trips, routes, and stops data in an open-source format that can be used for Google Transit Web-based trip planner. GTFS has evolved ever since based on the feedback from agencies and developers. To date, majority of transit agencies have made their GTFS data available to the general public (323 transit agencies nationwide) [59]. A GTFS dataset consists of several plain text files which have been formatted as Comma-Separated Values (CSV). In public transport network, stops represent transit stations where vehicles pick up and drop off passengers. Routes are sequence of two or more stops whose schedule is followed by a transit vehicle. Multiple trips can occur following the same route throughout a day. Therefore, a trip is a sequence of two or more stops that occurs at a specific time.

**Fig 1:**
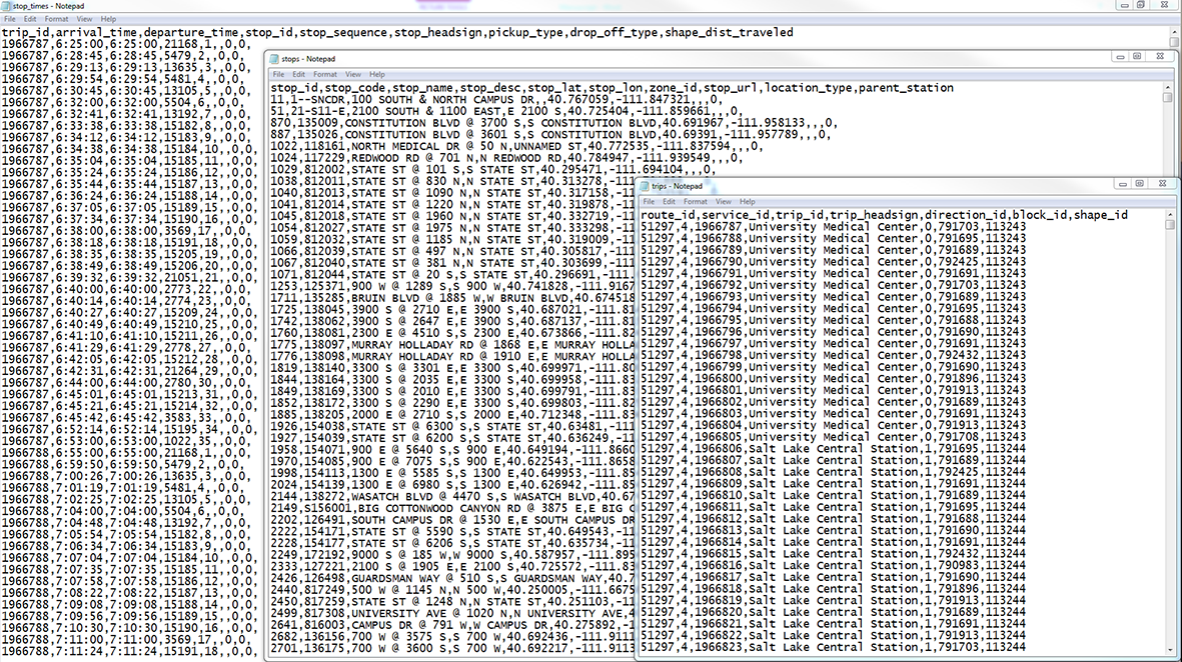
Stop-Times, stops, and trips files of GTFS for St. George, UT.

Fig 1 shows a snapshot of GTFS Stops, Trips, and Stop-Times for the City of St. George’s transit network. These files, combined with Calendar and Routes files contain detailed information about transit schedule for every minute of any day-of-week.

Previous studies presented examples on transit service evaluation using GTFS data, such as service area calculation, service coverage, time and distance service calculation, stop location and spacing optimization, service frequency, and span of service [60–62]. The scope of GTFS applications can be significantly expanded when combined with other datasets. For example, when jointly considered with Automatic Passenger Count (APC) data, GTFS can unveil important transit performance profile such as ridership-by-hour, by-trip, and by-stop, trip activity ranking, stop activity ranking, and activity-by-period. When combined with census data, GTFS might offer valuable information for transit connectivity and accessibility [12, 28, 63–65]. In this study, we will demonstrate such an example for travel time calculation using GTFS combined with census data.

## Methodology

**Fig 2:**
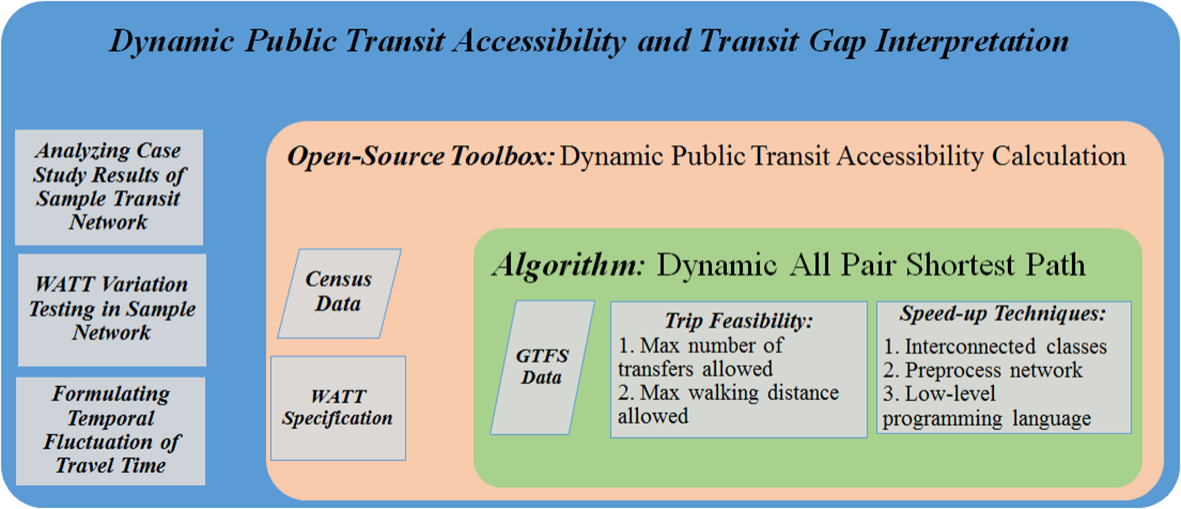
Methodological framework.

In this section, we will present our algorithm design for computing accessibility measures using GTFS data. The core component is the capability of finding the shortest path and updating the travel time between stations in both spatiotemporal dimensions. We further decipher the travel time matrix to explore the impact of network connectivity on accessibility measure. Fig 2 presents the overall methodological framework of this research including the core components (e.g. datasets, techniques, and formula) and their relations.

### Algorithm design

The proposed algorithm starts at each station by finding the next available trip passing through this station and the immediately connected stations at a specific time-of-day. These trips are further traced and travel times for stations met on these trips are updated. If the met stations are transfer stations (connected to a new route) then the next available trip on the new route is traced as well. This process continues until either all the stations in the network are met or the trips appear unpractical from users’ perspective. All the calculation of travel times are based on the time-table reading from GTFS files, so there is no need to build the network graph for each departure time. We assume that any user is willing to take up to four transfers and walk up to 700 meters for transfers to reach a destination [66]. With this assumption, the algorithm is described as follows:

#### Step 1: Input data

The GTFS data is read into three classes including stops, routes, and trips. Stop class contains route and trip members that stores the passing routes and trip IDs. The route class includes stop member storing station IDs that are visited by the routes. The trips are stored in hash tables in order to improve the process of finding the next available trips.

#### Step 2: Find connected routes to each station and update travel time by walking

In this step, the distances between all stations are calculated and converted to travel time assuming a constant walking speed of 2.98 miles per hour [67]. The values are stored in a stop class member vector called travel time (*tt_i_*). In addition, if a stop is in close vicinity of another stop within 700 meter radius that serves different routes, those routes will be added to route member and both stops will be added as connected stops member of stop class. The time complexity of this step is O (V^2^).

It is important to mention that when a destination was not reachable within four transfers, the walking time between origin and destination stations was selected as the travel time. This prevents the WATT value from becoming extremely small or large considering the numerator in Eq (3). Specifically, the impact of travel time to reachable destinations will be undermined if a large travel time value is selected for non-accessible destinations. The walking time is used as travel time between origin and destination stations only in cases where transit travel time is longer than walking time and walking distance is less than 700 meters.

#### Step 3: Find all-pairs travel time and WATT for each station for all time-of-day

**Fig 3:**
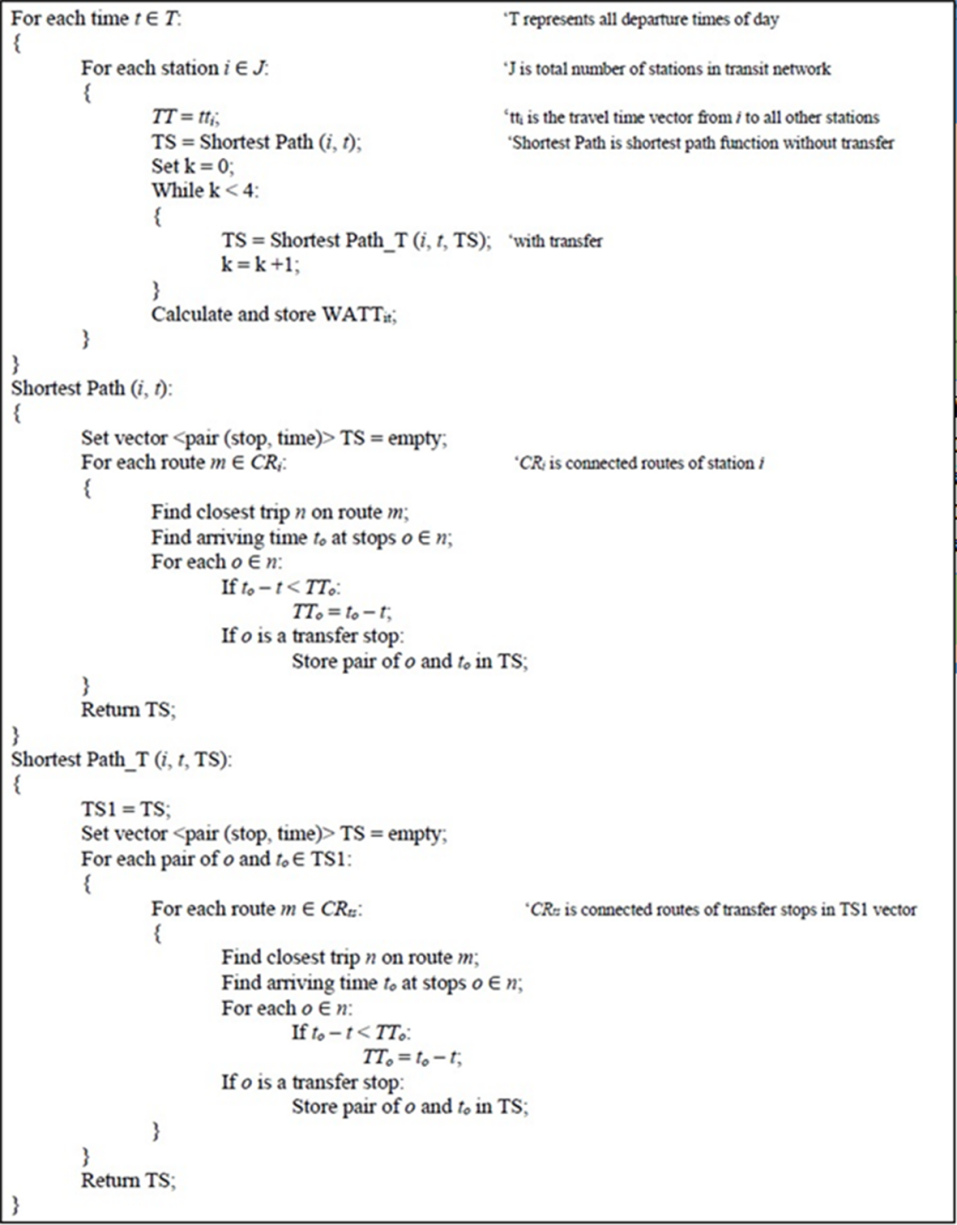
Pseudo-code for finding all-pairs shortest path and station WATT through a day.

The pseudo-code for calculating all-pairs travel time for all time-of-day is shown in Fig 3. In the pseudo-code *k* represents the number of transfers allowed, *shortest path* function finds and updates the travel time from stop *i* (origin) to other stops that are connected to stop *i* without transfer, *shortest path T* function finds and updates the travel time from stop *i* (origin) to other stops that are connected to stop *i* with 1, 2, 3, and 4 transfers, respectively in each *k while loop, t_o_* represents the earliest time to arrive at stop *o* from stop *i* and it is directly read from trips class, *t* represents the departure time from stop *i*, and *t_o_−t* is the shortest path (travel time) from stop *i* to stop *o*.

As shown in Fig 3, the time complexity of proposed algorithm in Step 3 is *O*(*T* * *V* * *R* * *S*), where *T* represents number of time intervals, *V* is the total number of stations, *R* is the average number of routes available within four transfers of stations, and *S* is the average number of stations on each route. *S*−1 excludes the selected origin station from *S*. The time complexity of the algorithm then becomes *O*(*V*^2^ + *T* * *V* * *R* * *S*). As the number of time intervals increases, *V*^*2*^ becomes negligible comparing with *T* * *V* * *R* * (*S*−1). Therefore, the time complexity is nearly *O* (*TVRS*).

#### Time complexity comparison

As mentioned in the *Literature Review* section, the fastest algorithm for time discrete all-pairs shortest path available to date was introduced by Pettie [44] and has a time complexity of *O* (*EV* + *V*^2^ *log* (*log* (*V*))). Implementing Pettie’s algorithm onto time-dependent network will result in time complexity of *O* (*TEV* + *TV*^2^ *log* (*log* (*V*))). In order to compare the time complexity of proposed algorithm with Pettie’s algorithm, the *O* (*TVRS*) must be compared with *O max* (*TEV*, *TV*^2^ *log* (*log* (*V*))). As a result, if one of the statements in Eqs (4) and (5) holds true, then our proposed algorithm have lower time complexity than Pettie’s.

TVRS≤TEV→RS≤E (4)

$$TVRS\leq TV^{2}\;\log\left(\log\left(V\right)\right)\to RS\leq Vlog(\log\left(V\right))$$ (5)

Proof of Eq (4):

*R* represents the average number of routes available within four transfers of stations. Consequently, the maximum value for *R* equals to the total number of available routes in the network. *E* is the total number of routes connecting two consecutive stops and *E* = *R*_*max*_ * *S*. Thus, *R* * *S* ≤ *E* always holds true. It is therefore evident that the time complexity of the proposed algorithm is lower than Pettie’s. As the transit network size grows, the ratio of $\frac{R_{max}}{R}$ will increase. This indicates that with the increase of number of nodes and edges in time dependent all-pairs shortest path problem, the time complexity of Pettie’s algorithm increases at a faster rate than that of our proposed algorithm. The computational performance is verified in the *Application* section where the algorithm is tested on an actual transit network.

### WATT

As mentioned in *Literature Review*, WATT is an accessibility measure that weights travel time based on the potential opportunities of destinations. Since the potential opportunities available at each destination is assumed constant throughout the day, a closer look of travel time matrix can provide valuable insights on WATT fluctuation. Assume *k*_0*i*_, *k*_1*i*_, *k*_2*i*_, *k*_3*i*_, *and k*_4*i*_ represent sets of stations (destinations) reachable from station *i* by taking 0, 1, 2, 3, and 4 transfers, respectively. *k*_*non–reachable i*_ is the set of stations that cannot be reached by taking 4 transfers from station *i*. Then the travel time from station *i* to any given station in *k*_0*i*_ at time-of-day *t* will be:

$$tt_{ij_{t}}=WT_{0_{t}}+\frac{d_{ij}}{OS_{0}},\;j\;\epsilon \;k_{0i}$$ (6)

where $tt_{ij_{t}}$ is the travel time from station *i* to *j* at time *t*, $WT_{0_{t}}$ is the waiting time at station *i* for the connecting route at time *t*, *d*_*ij*_ is the distance between station *i* and *j*, and *OS*_0_ is the operating speed of the connecting route. Similarly, the travel time from station *i* to any given station in *k*_1*i*_ at time-of-day *t* becomes:

$$tt_{ij_{t}}=WT_{0_{ct}}+\frac{d_{ic}}{OS_{0c}}+WT_{1_{t+ct}}+\frac{d_{cj}}{OS_{1}},\;j\;\epsilon \;k_{1i}$$ (7)

where *c* represents transfer station on travel routes from *i* to *j*, $WT_{0_{ct}}$ is the waiting time at station *i* for the connecting route between *i* and *c* at time *t*, *d*_*ic*_ is the distance between station *i* and *c*, *OS*_0*c*_ is the operating speed of the connecting route between *i* and *c*, $WT_{1_{t+ct}}$ is the waiting time at station *c* for the connecting route between *c* and *j* at time *t+ct*, *d*_*cj*_ is the distance between station *c* and *j*, and *OS*_1_ is the operating speed of the connecting route between *c* and *j*. *ct* is the travel time from station *i* to *c*, and is equal to $WT_{0_{ct}}+\frac{d_{ic}}{OS_{0c}}$.

Eq (7) can be further expanded as follows to find the travel time from station *i* to any given station in *k*_2*i*_, *k*_3*i*_, *and k*_4*i*_:

$$tt_{ij_{t}}=WT_{0_{c_{1}t}}+\frac{d_{ic_{1}}}{OS_{0c_{1}}}+WT_{1_{t+c_{1}t}}+\frac{d_{c_{1}c_{2}}}{OS_{1c_{2}}}+WT_{1_{t+c_{1}t+c_{2}t}}+\frac{d_{c2j}}{OS_{2}},\;j\;\epsilon \;k_{2i}$$ (8)

$$tt_{ij_{t}}=WT_{0_{c_{1}t}}+\frac{d_{ic_{1}}}{OS_{0c_{1}}}+WT_{1_{t+c_{1}t}}+\frac{d_{c_{1}c_{2}}}{OS_{1c_{2}}}+WT_{1_{t+c_{1}t+c_{2}t}}+\frac{d_{c_{2}c_{3}}}{OS_{2c_{3}}}+WT_{1_{t+c_{1}t+c_{2}t+c_{3}t}}+\frac{d_{c_{3}j}}{OS_{3}},\;j\;\epsilon \;k_{3i}$$ (9)

$$tt_{ij_{t}}=WT_{0_{c_{1}t}}+\frac{d_{ic_{1}}}{OS_{0c_{1}}}+WT_{1_{t+c_{1}t}}+\frac{d_{c_{1}c_{2}}}{OS_{1c_{2}}}+WT_{1_{t+c_{1}t+c_{2}t}}+\frac{d_{c_{2}c_{3}}}{OS_{2c_{3}}}+WT_{1_{t+c_{1}t+c_{2}t+c_{3}t}}+\frac{d_{c_{3}c_{4}}}{OS_{3c_{3}}}+WT_{1_{t+c_{1}t+c_{2}t+c_{3}t+c_{4}t}}+\frac{d_{c_{4}j}}{OS_{4}},\;j\;\epsilon \;k_{4i}$$ (10)

where indices *c*_1_, *c*_2_, *c*_3_, *and c*_4_ represent first, second, third, and fourth transfer stations, respectively. Assuming that operating speed of routes does not change throughout the day, then the average travel times from station *i* to any other station become:

$$Average\;tt_{ij}=\frac{\sum_{t=0}^{T}WT_{0_{t}}}{n}+\frac{d_{ij}}{OS_{0}},\;j\;\epsilon \;k_{0i}$$ (11)

$$Average\;tt_{ij}=\frac{\sum_{t=0}^{T}WT_{0_{ct}}+WT_{1_{t+ct}}}{n}+\frac{d_{ic}}{OS_{0c}}+\frac{d_{cj}}{OS_{1}},\;j\;\epsilon \;k_{1i}$$ (12)

$$Average\;tt_{ij}=\frac{\sum_{t=0}^{T}WT_{0_{c_{1}t}}+WT_{1_{t+c_{1}t}}+WT_{1_{t+c_{1}t+c_{2}t}}}{n}+\frac{d_{ic_{1}}}{OS_{0c_{1}}}+\frac{d_{c_{1}c_{2}}}{OS_{1c_{2}}}+\frac{d_{c2j}}{OS_{2}},\;j\;\epsilon \;k_{2i}$$ (13)

$$Average\;tt_{ij}=\frac{\sum_{t=0}^{T}WT_{0_{c_{1}t}}+WT_{1_{t+c_{1}t}}+WT_{1_{t+c_{1}t+c_{2}t}}+WT_{1_{t+c_{1}t+c_{2}t+c_{3}t}}}{n}+\frac{d_{ic_{1}}}{OS_{0c_{1}}}++\frac{d_{c_{1}c_{2}}}{OS_{1c_{2}}}++\frac{d_{c_{2}c_{3}}}{OS_{2c_{3}}}++\frac{d_{c_{3}j}}{OS_{3}},\;j\;\epsilon \;k_{3i}$$ (14)

$$Average\;tt_{ij}=\frac{\sum_{t=0}^{T}WT_{0_{c_{1}t}}+WT_{1_{t+c_{1}t}}+WT_{1_{t+c_{1}t+c_{2}t}}+WT_{1_{t+c_{1}t+c_{2}t+c_{3}t}}+WT_{1_{t+c_{1}t+c_{2}t+c_{3}t+c_{4}t}}}{n}+\frac{d_{ic_{1}}}{OS_{0c_{1}}}+\frac{d_{c_{1}c_{2}}}{OS_{1c_{2}}}+\frac{d_{c_{2}c_{3}}}{OS_{2c_{3}}}+\frac{d_{c_{3}c_{4}}}{OS_{3c_{3}}}+\frac{d_{c_{4}j}}{OS_{4}},\;j\;\epsilon \;k_{4i}$$ (15)

where *n* is the total number of departure times (*t*) that travel time is calculated between *i* and *j*, and *T* is the last departure time. As it shows in Eqs (6) through (15), the fluctuation on travel time from station *i* to other stations is predominantly determined by the waiting time variation at different times-of-day. Each additional transfer taken to reach destinations will add an extra transfer waiting time and in-vehicle time. Thus, the stations that are connected to rather higher number of routes (more stations in set *k*_0*i*_) are likely to have lowered average travel time to destinations. In addition if the stations in set *k*_0*i*_ have relatively higher potential opportunities available, then station *i* would have better WATT. For an average station *i*, the size of sets *k*_0*i*_, *k*_1*i*_, *k*_2*i*_ are smaller in low connectivity network than highly connected networks. This shows the importance of route (network) connectivity on network travel time and accessibility.

## Application

The algorithm developed in this paper is tested onto the City of St. George transit network operated by SUNTRAN in the State of Utah. In this section, we present a brief description of SUNTRAN transit network, followed by algorithm efficiency assessment and transit accessibility results.

### Study network: SUNTRAN network

**Fig 4:**
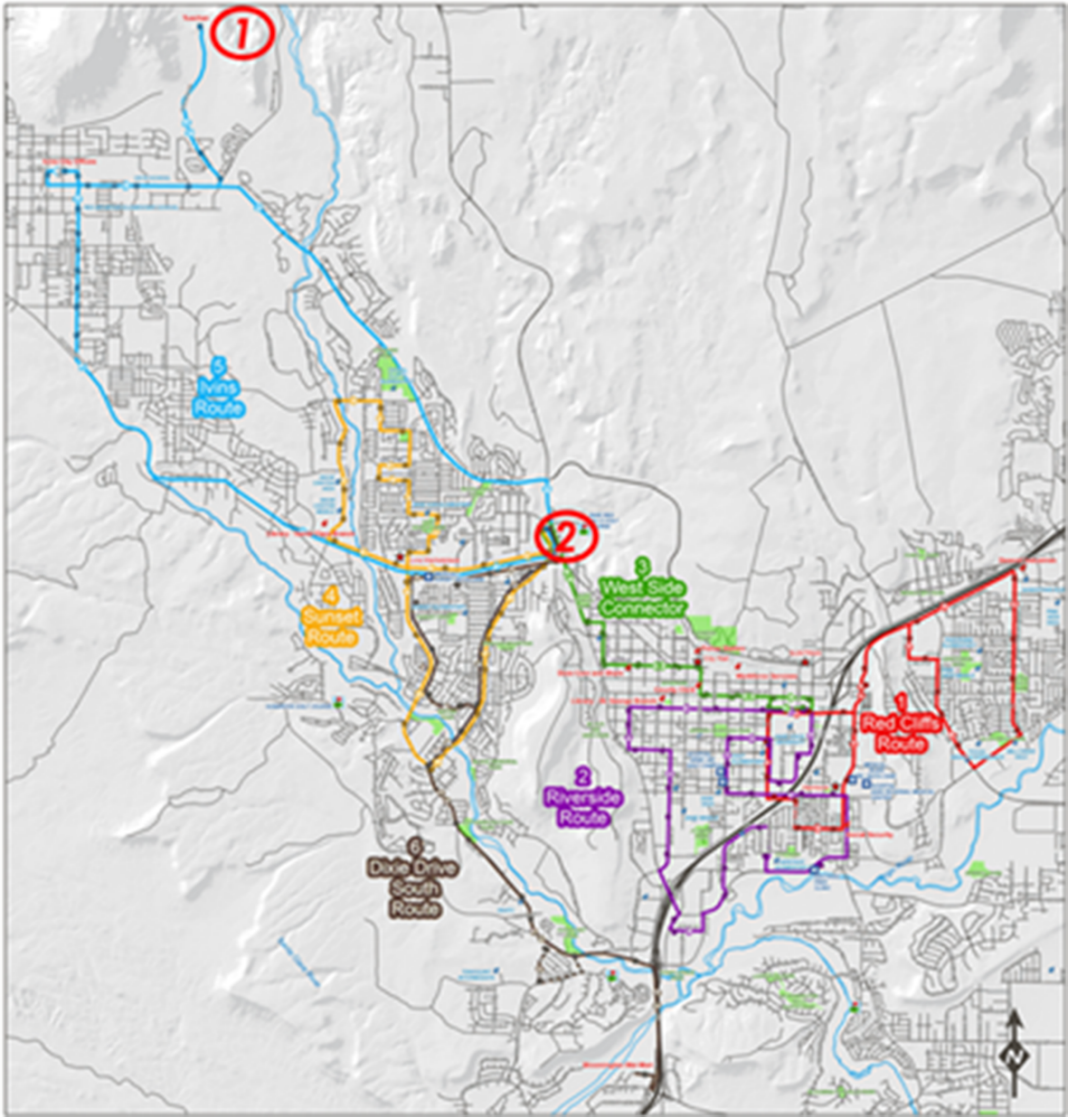
SUNTRAN’s transit network map; *1*: *Taucahn Station*, *2*: *Sunset Corner Station*.

St. George is a small-sized city located in Southern Utah with a population of 76,817. Census block data for the State of Utah in 2015 have been collected from the Utah Automated Geographic Reference Center (AGRC) website [68] also available in S1 Dataset. Census block is the smallest geographic unit used by United States Census Bureau for tabulation of data collected from all houses. The high resolution of census block data ensures the accuracy of accessibility measures. The data were used to calculate the population within a 700-meter radius of each transit station to represent the potential opportunity offered by that station. The GTFS data for St. George transit network operated by SUNTRAN were retrieved from GTFS data exchange website [69]. SUNTRAN’s transit network consists of six bus routes and 134 bus stops. Four of these routes operate at 40-minute fixed headway and the other two operate at 80-minute fixed headway. Fig 4 shows SUNTRAN’s transit network map. The two stations highlighted in Fig 4 are used for micro-level analysis of transit accessibility.

Other than using the population density within 700-meter radius of stations to represent the potential opportunity, all-pairs shortest paths for each time-of-day are needed to calculate the travel time component of WATT. This is completed using the GTFS data via the developed algorithm presented in Fig 3.

### Algorithm efficiency

To validate the computational efficiency of the proposed algorithm, we compared our algorithm against Pettie’s algorithm. Both algorithms for time dependent all-pairs shortest path search were coded in C++. In addition, the computation time using Esri’s ArcMap Network was estimated. All calculations were performed using a desktop computer with an Intel® Core™ i7-4790 3.6 GHz computer processor and 16 GB of RAM. The results of this computation experiment are shown in Table 1. It indicates that even for a relatively small-sized network where *R* ∼ *Rmax*, the proposed algorithm outperforms Pettie’s algorithm. This is probably due to the necessity of rebuilding the network graph for each time interval as an additional step in Pettie’s algorithm. The Esri’s ArcMap Network Analyst, the most commonly used commercial software package for spatial analysis, is more than three times slower than our proposed algorithm. We expect that the proposed algorithm perform even better as the network size grows and are exploring it in an ongoing study. The algorithm was implemented in Fayyaz et al. [64] to compute dynamic transit accessibility for the entire Utah Transit Authority’s (UTA) network. UTA’s network consists of 6,265 transit stations and 125 transit routes including commuter rail, light rail, bus rapid transit, and bus. The WATT was measured for all stations every 10-minute interval from 4 AM to 10 PM (4,239,024,300 unique shortest paths). Fayyaz et al. [64] has reported that the total computation time was less than 6 days whereas the same calculation in Esri’s ArcGIS would take up to 165 days.

**Table 1:** Computational time for time dependent all-pairs shortest paths in SUNTRAN’s transit network.

***SUNTRAN transit network*, *St*. *George***	
No. of stations	134
No. of routes	6
No. of Origin-destination pairs	17822
***WATT computation time (for five minute interval from 5 AM to 8 PM)***	
Uniquely computed shortest path = 17822 * 181 = 3,*225*,*782*	
Proposed algorithm	210 Seconds
Pettie's algorithm	273 Seconds
Esri ArcMap Network Analyst	672 Seconds

### WATT results and transit accessibility analysis

**Fig 5:**
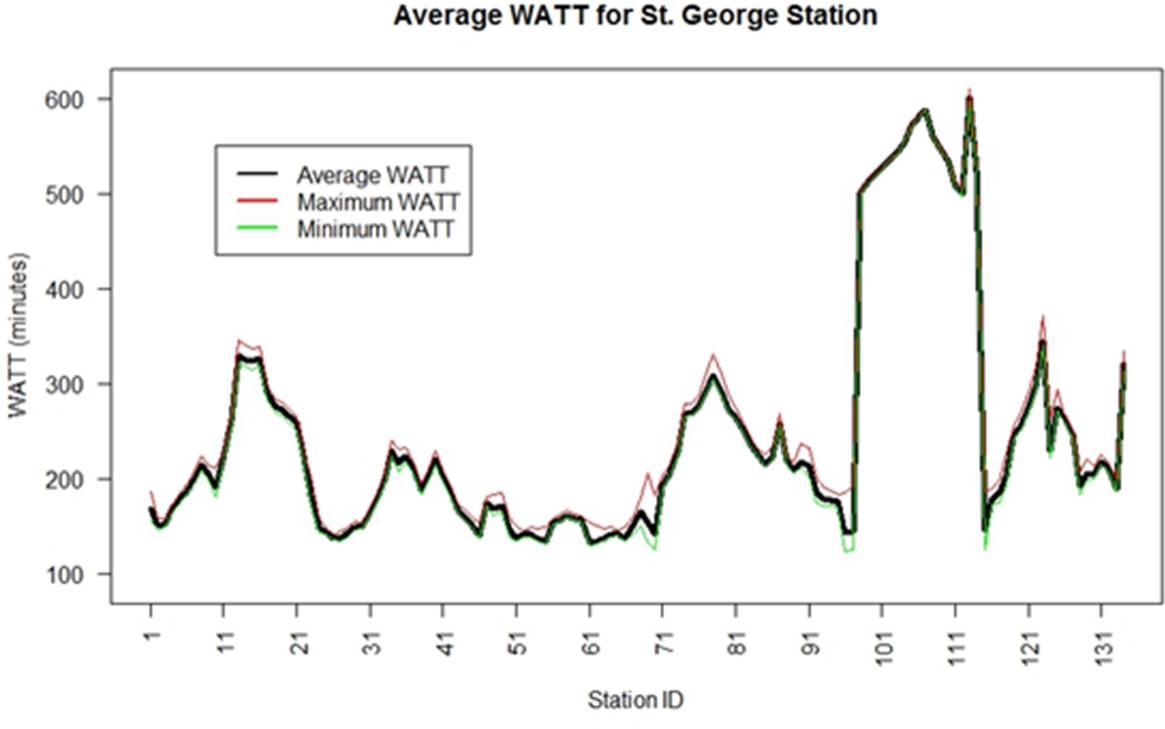
WATT plotted regarding station ID.

WATT was calculated for all stations for every 5-minute interval from 5 AM to 8 PM throughout a typical weekday. Fig 5 shows the average, maximum, and minimum WATT result for each station within the network. Note that the WATT values are conglomerated based on station ID, with high WATT representing low accessibility and vice versa. This is due to the fact that station IDs are labeled sequentially for stations along the same route in St. George’s network.

**Fig 6:**
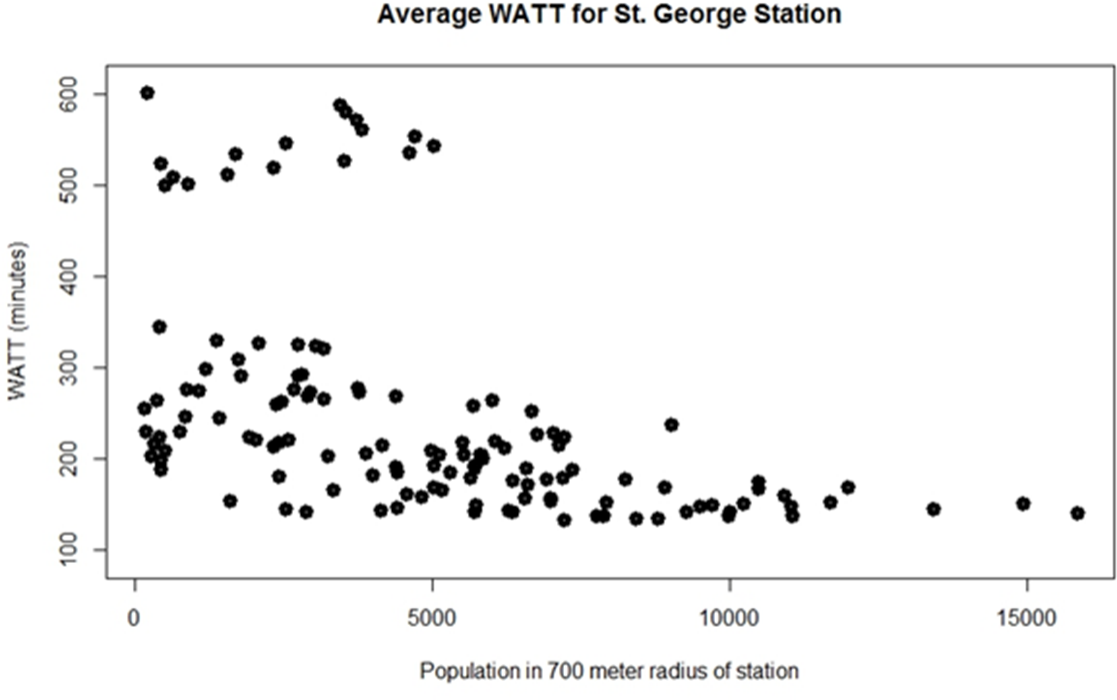
WATT plotted regarding the station population living in 700 meter radius.

WATT was further plotted against the population within 700-meter radius of each station and the result is shown in Fig 6. A station that have low accessibility value (a.k.a. high WATT) and low population in the vicinity does not raise too much concern. Yet problem arises at stations with high accessibility (a.k.a. low WATT) and low population or at stations with low accessibility (a.k.a. high WATT) and high population. In the first scenario, redundancy exists in the current transit network with very scarce demand which results in network inefficiencies and waste of resources. In the second scenario, demand exceeds supply leading to customer dissatisfaction and inequitable access. The stations fall into the second scenario are highlighted with red circle in Fig 6. These stations should be given top priority for improved transit service. The stations with excess supply (first scenario) are highlighted with green circle. These stations must be studied under a closer scrutiny to further justify their existence. Removal of these stations might be necessary in case of budget constraint. To better capture the equity issue, socioeconomic characteristics of the population around the stations can be considered for a better result filtration. Unfortunately, for the City of St. George, the variation of socioeconomic characteristics (i.e. age, gender, average salary) across different transit stations is insignificant, impeding the equity analysis.

**Fig 7:**
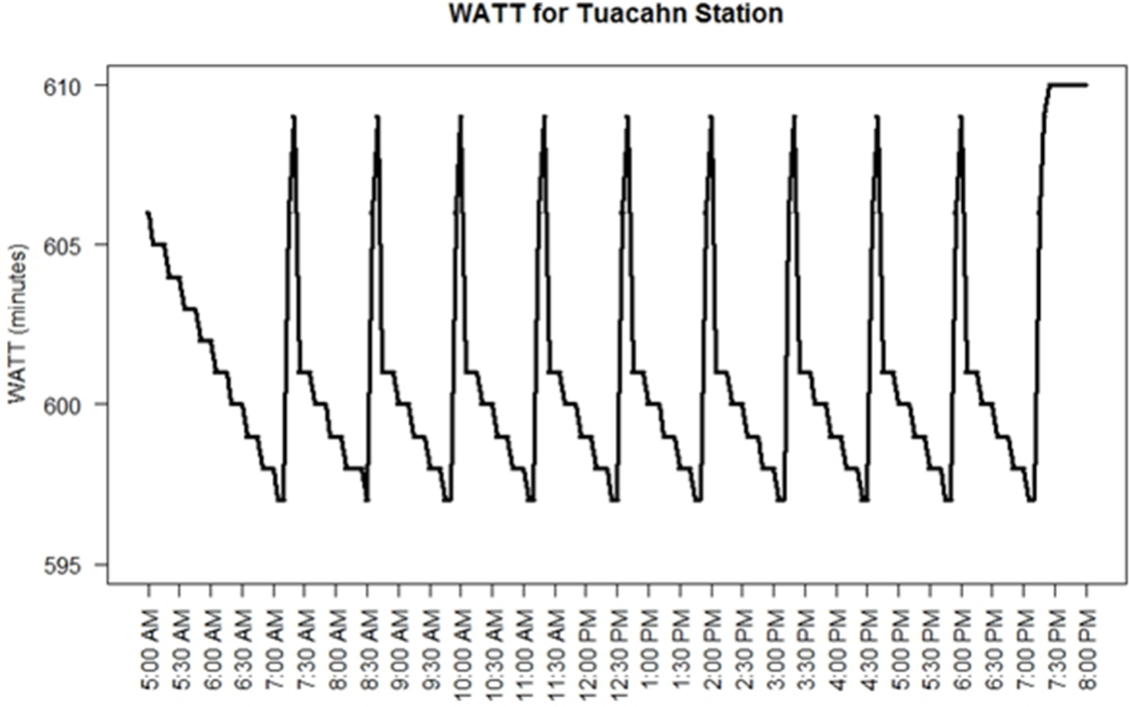
Temporal fluctuation in WATT for Tuacahn station.

**Fig 8:**
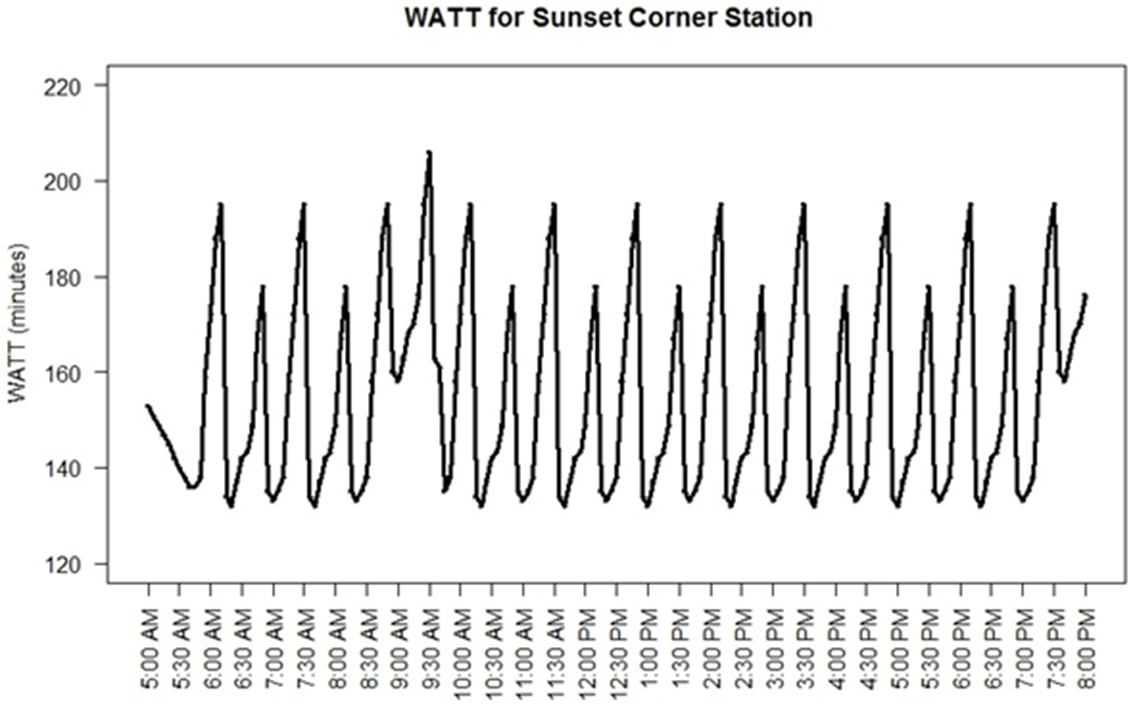
Temporal fluctuation in WATT for Sunset Corner station.

To better illustrate the temporal fluctuation of WATT, two stations were chosen for micro-level analysis, including Tuacahn station and Sunset Corner station with highest and lowest WATT values respectively (Figs 7 and 8). Tuacahn station (marked in Fig 4) is located in recreational area with a population of 20 people living within the 700-meter radius. Bus route 5 is the only bus serving the station that operates on an 80-minute headway. On the contrary, Sunset Corner station is located close to shopping centers and residential areas with 1,600 people living within the 700-meter radius. Bus routes 3, 4, 5, and 6 are serving this station. As shown in Fig 7, due to its geographical remoteness and scarce transit service, the accessibility of the Tuacahn station reaches its maximum (lowest WATT) when the bus is at the station and goes back to minimum right after it departs (highest WATT). Transit accessibility then will gradually increase as the next bus is approaching. The time interval between the two consecutive peak points is equal to 80 minutes (operating headway of the serving route). The accessibility of the station would not be improved significantly (with a marginal 10 to15-minute difference in WATT) even when the bus is at station due to its remote location. On the other hand, Sunset Corner station is located in close vicinity of the city center and served by four transit routes. Fig 8 shows that the time interval between the two consecutive peak points for this station varies between 15 to 30 minutes. This difference is dependent on the coordination between the four routes serving the station. Fig 8 indicates that the coordination between the four routes at Sunset Corner station is at worst condition around 9:35 AM. This is due to the unavailability of service from Route 5 at that hour for the station.

**Fig 9:**
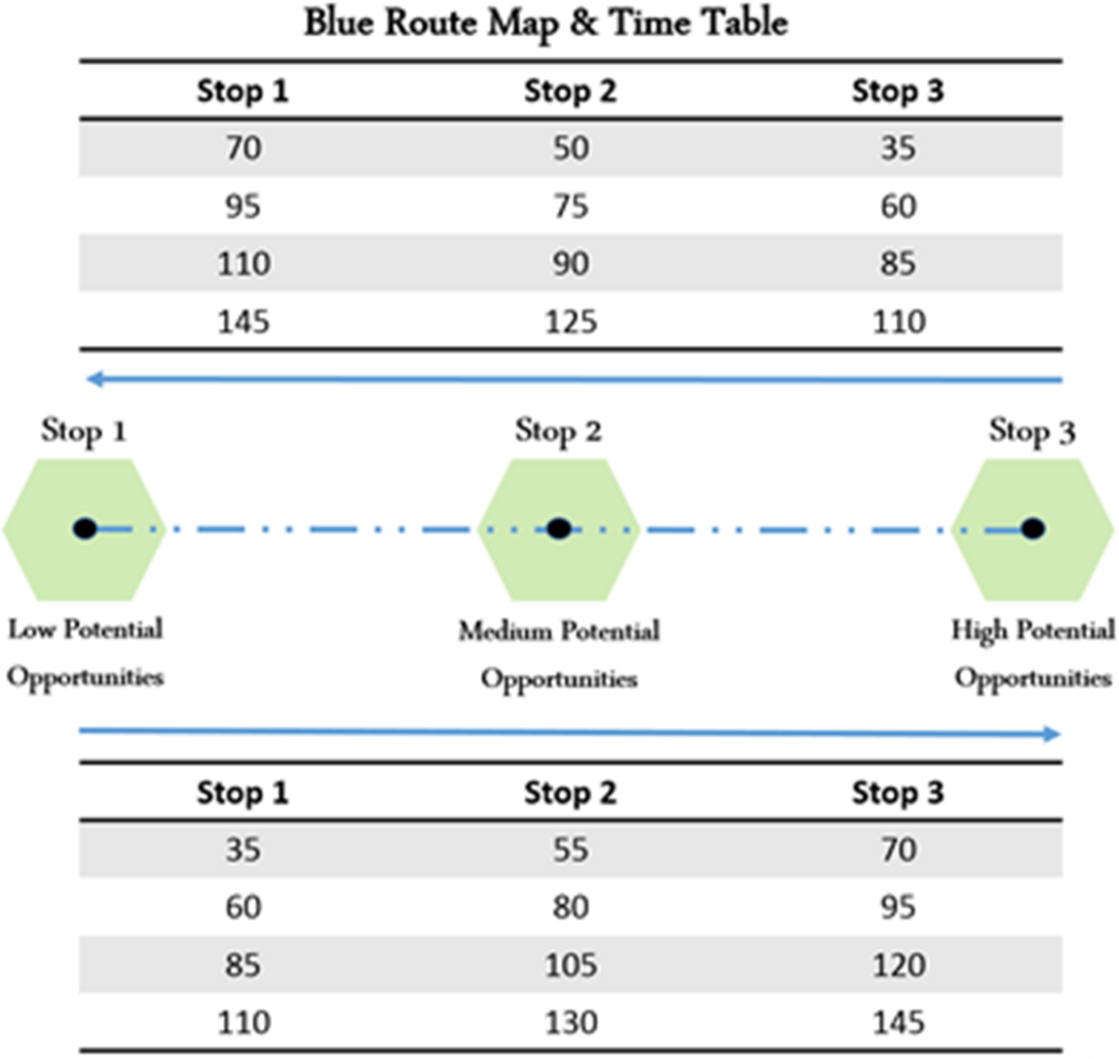
Representation of a simple transit network.

To better interpret WATT’s implications, we present a simple network consisting of one transit route and three transit stations. The route is operating on a 25-minute headway. Route’s timetable is shown in Fig 9 representing route departure time at each station and direction. The impact of station’s geographical location can be captured by comparing WATT of the three stations, given that all of them are served by the same route.

**Fig 10:**
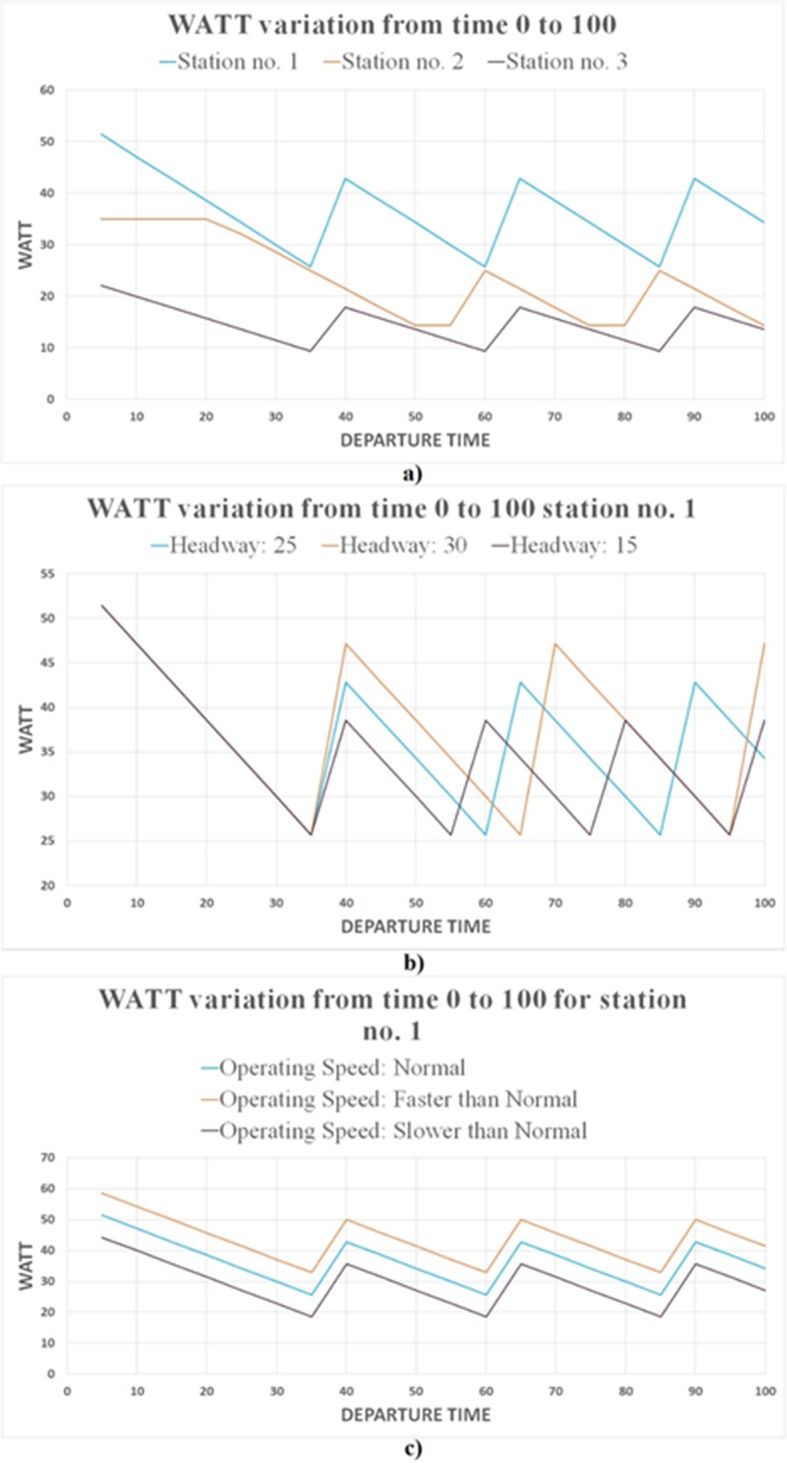
WATT variation throughout the day. (A) Stations no. 1, 2, and 3, (B) different headways, (C) different operating speed.

Fig 10A shows the WATT variation throughout the day for all three stations. Station No. 1 has the worse WATT since it requires the longest distance (travel time) to reach potential opportunities. The poor accessibility of Station No. 1 is caused by geographical disadvantage, which is demonstrated in the higher WATT values compared to Stations No. 2 and No. 3. Fig 10B shows the sensitivity analysis of WATT for Station No. 1 in response to changing headways. Note that since only one route is in service, the waiting time will always increase abruptly the moment that bus departs from the station, which is manifested in Fig 10B as pulses (WATT changes from minimum to maximum). Also note that the decreasing slope of WATT is always constant. This is caused by gradual reduction in waiting time and consequently gradual reduction in travel time and WATT. Thus, the route headway will determine the time lag between local minimum and maximum WATTs, and consequently, the range of WATT fluctuation. Fig 10C shows the varying effects of route’s operating speed on WATT. The headway is kept same (25-minute), and consequently the time lag between local WATT maximum and minimum is the same across all three scenarios. The operating speed, as can be observed, directly affects the local minimum WATT. Faster service shortens travel time, thus people can reach their destinations quicker, and the local minimum WATT (when waiting time is zero) decreases.

**Fig 11:**
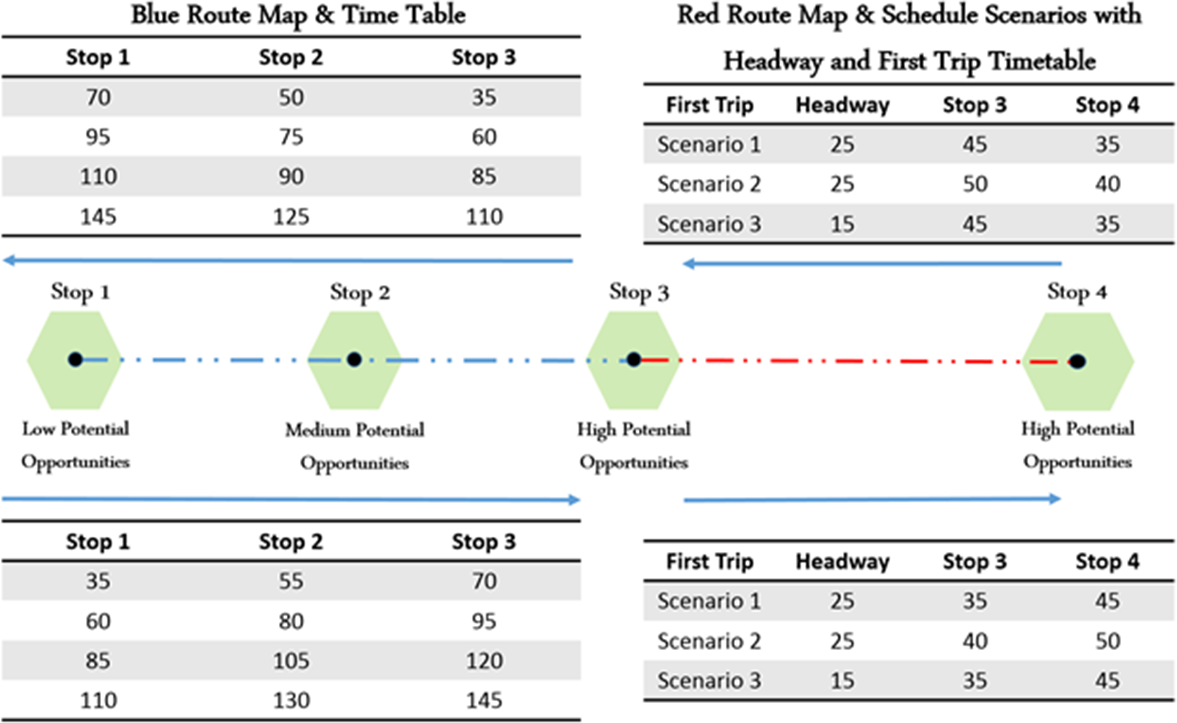
Simplified network with two routes.

**Fig 12:**
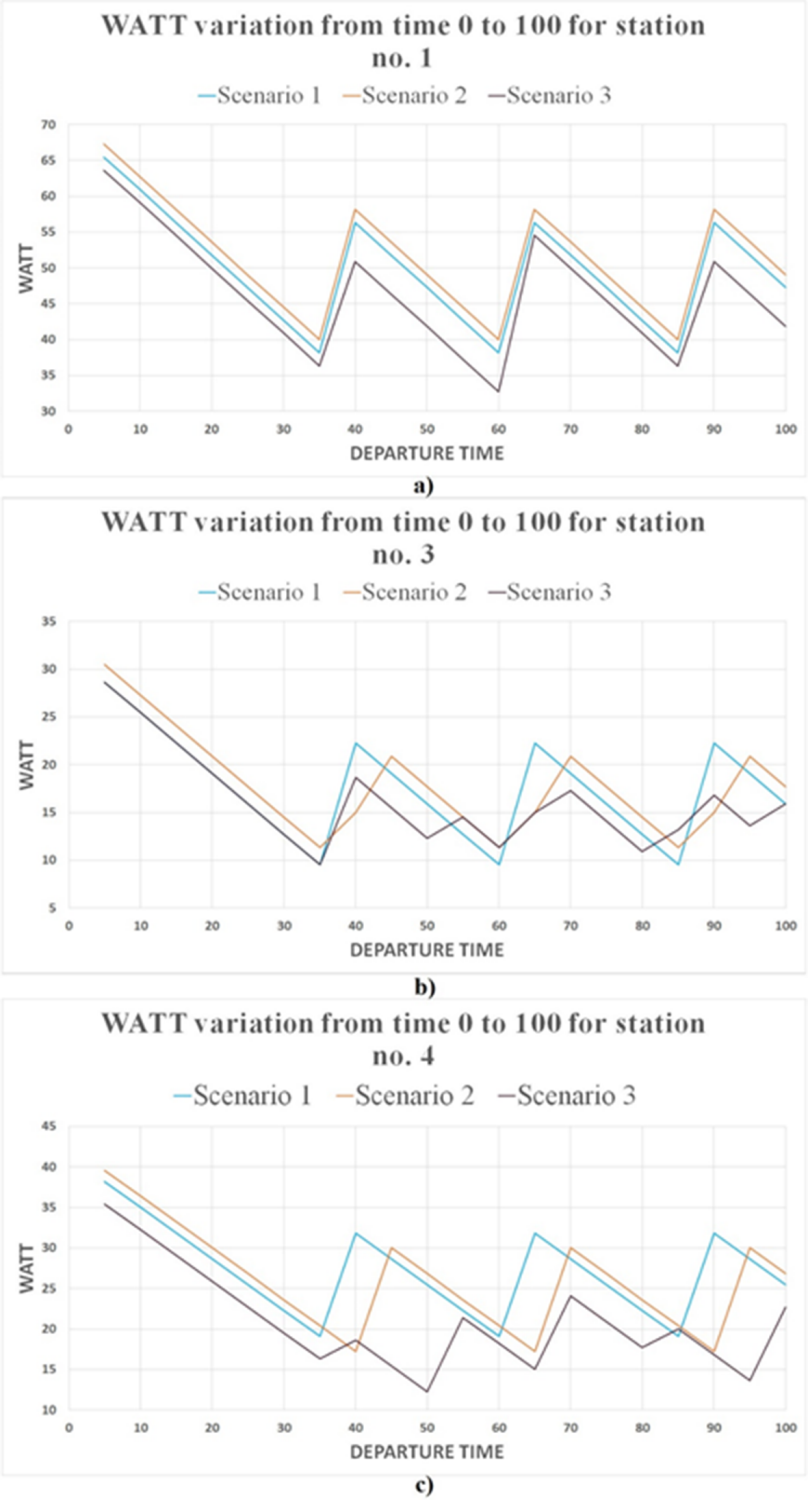
WATT variation throughout the day in three scenarios. (A) Station no. 1; (B) station no. 3; and (C) station no. 4.

Building upon the sample network shown, an extra station (Station No. 4) and an extra route (Red route) are added and shown in Fig 11. Three different scenarios were developed to demonstrate the influence of incoordination and headway differences between Blue and Red routes on Stations No. 1, 3, and 4’s WATT. In scenario 1, both routes are operating on the same headway (25 minutes) and same initial bus departure time. In scenario 2, both routes are operating on the same headway (25 minutes) but the first bus of Red route departs 5 minute later than the first bus of Blue route. In scenario 3, the initial bus departure time of both routes are the same, but their headways differ (25 minutes for Blue route and 15 minutes for Red route). The different departure time in scenario 2 leads to 5-minute more waiting time for traveling from Station No. 1 to Station No. 4 comparing with scenario 1. This can be seen in Fig 12A as the WATT difference (y-axis) between the two scenarios. However, this departure time difference has a positive effect on WATT of Station No. 4 (Fig 12C), since it enables faster access to Station No. 3 and have no effect on waiting time to reach Stations No. 1 and 2. Thus, the incoordination of routes increases travel time and WATT in one direction and decreases travel time and WATT in other direction. This also indicates that there exists an optimum coordination which will minimize the sum of WATTs throughout the day on both directions.

Finally, the impact of headway differences between Blue and Red routes is reflected in scenario 3. Comparing scenario 3 with scenarios 1 and 2, it shows that lower headway of red route generally decreases travel time and WATT throughout the day. However, the extent of reduction on WATT varies for different stations. Station No. 4 is experiencing the highest reduction in WATT. Station No. 4 relies on Red route to reach all other stations in the network. As a result, lower headway of Red route will result in lower wait time and WATT. Note that only scenario 3 demonstrates the inconsistence in local minimum/maximum WATT, due to the varying waiting times for transfer between routes. The WATT graphs can be used to adjust the transit service coordination such that the local minimum WATT can occur when the demand is high.

Based on the above analysis, the WATT pattern shown for the St. George network can be better deciphered. Revisiting Figs 7 and 8, it shows that Tuacahn station is geographically disadvantaged as demonstrated by the high global maximum WATT. The long headway (80 minutes) of Tuacahn station (Fig 7) leads to the large range of WATT, as a result the local maximum WATT almost reaches the station’s global maximum. The operating speed of the serving route is also rather slow since the local minimum WATTs are only 13 minutes less than global maximum WATT. There is no headway differences in the network as all the routes are operating at 40 or 80 minute fixed headway. Thus, there is no differences between local maximum (minimum) WATTs.

The headway for Sunset Corner station (Fig 8) is affected by arrival time of buses operating on the several routes serving this station. As a result, the time lag between to local maximum (minimum) WATTs is shorter than 40 minutes due to the overlapping effect. Sunset Corner station’s WATT variation has a similar pattern as Fig 12B scenario 2. In such cases, the local maximum WATTs are not always reached momentarily due to bus departure. The local minimum WATTs are consistent throughout the day indicating that the routes serving the station are providing almost the same operating speed to potential destinations since different local minimums are caused by different routes (or combination of them). The local minimum WATTs are much smaller than global maximum WATTs in Fig 8 (in order of 85 minutes), indicating that the serving routes (or combination of them) are operating on rather high speeds.

The WATT pattern of stations in St. George network is repetitive throughout the day. This is caused by the fact that limited number of routes (6) are operating on fixed headways that are 40-minutes or its multiple (80 minutes). In bigger networks, the WATT pattern analysis will become more complex and it can better show temporal deficiencies of service. We believe the station-level WATT graph is a powerful tool for public transit agencies and planners in conducting microscopic transit performance analysis. It not only captures transit service performance measures such as headway, operating speed, coordination, and travel time, but also associates them with land use and potential opportunities available (geographic distribution of attractiveness).

## Conclusion

Public transit accessibility has been studied for many years in evaluation of transit services. However, the time-dependent transit accessibility has not been explored much until recently, due to the unavailability of transit schedule data. The introduction of GTFS greatly facilitates such analysis by offering high resolution transit schedule data in a standard format.

Existing research acknowledged that transit accessibility measures that capture the spatiotemporal patterns are most helpful for analyzing the gap in transit services. Yet the computational efficiency is challenging for all the OD pairs within the transit network. We introduce an efficient and innovative algorithm to solve this issue and prove that it has lower time complexity (*O* (*V*^2^ + *TVR* (*S*−1))) compared to the fastest shortest path algorithm to date (O (TEV + TV^2^ log (log (V))). The proposed shortest path algorithm limits the number of transfers for each transit trip to four. This constraint not only makes the result more realistic but also improves time complexity and speed of the algorithm significantly.

The proposed algorithm is implemented in C++ and utilizes the publically available datasets including GTFS and Census data. The methodology is applied to SUNTRAN’s transit network in City of St. George, UT. The result shows that our proposed algorithm outperforms other widely used all-pairs shortest-path algorithms/packages, including Petti’s and Esri’s ArcMap Network Analyst. This difference will grow exponentially as network expands since the difference between the number of routes connected within 4 transfers will become much less than total number of routes of the network. For example, a simple estimation of computation time for a large-size network with 6,200 stops and 125 routes for every 10-minute of the day from 4 AM to 10 PM is approximately 5.2 days for our proposed algorithm and 165 days for Esri’s ArcMap Network Analyst. Another contribution of this paper is the introduction of the analysis method for interpreting transit accessibility. The macro-level analysis on WATT is effective in identifying stations with excessive supply or demand. The micro-level analysis can be used for tracking the temporal fluctuation of accessibility and identifying inefficiencies in bus coordination/scheduling.

Future research will focus on incorporating socioeconomic characteristics, travel patterns, and apply the methodology to a larger network. For example, the average income of population living in the vicinity of transit stations can be used as a surrogate for prioritizing public transit investments. The potential opportunities component of WATT aims to weight travel time based on the attractiveness of the destinations. Such weighing function and consequently the PTA results can be further enhanced by considering the travel pattern of transit users [70–72]. Implementing the framework to a larger network (more routes and stations) with both fixed and variable headway routes will provide better insights on the data management capability of the toolbox and showcase the temporal trends in network coordination. Finally, future expansions of the algorithm can benefit from adding several multimodal options for ingress and egress to transit stations such as park-and-ride and cycling.

## Supporting information

S1 Dataset GTFS for upload. (ZIP)
